# External Application of the Oil of Ergot

**Published:** 1841-02

**Authors:** 


*External Application of the Oil of Ergot.*—Mr. Wright has found this oil a valuable external application in cases of local rheumatism. He has tried it in three instances, and in all of them it has proved curative. The affected part should be well rubbed with it for a quarter of an hour, night and morning, until relief be obtained. It is one or the best remedies with which he is acquainted for the cure of toothache.

He has repeatedly known it to subdue the pain when kreosote has failed. But perhaps its greatest value, as an external application, is in the arresting of hæmorrhage. He has often wounded small arteries in dogs and rabbits, and subdued the bleeding with a drop of this oil. Hæmorrhage from the jugular and femoral veins has been similarly arrested. The troublesome bleeding which sometimes follows the extraction of a tooth, and leech-bites, it is equally efficient in stopping.

In a severe case of epistaxis, he arrested the hæmorrhage, by injecting up the nostrils equal parts of very dilute spirit and oil of ergot; and he has little doubt that in the severe cases of flooding which succeed delivery, the injection of this oil diffused through water into the uterus would be productive of the happiest results.—*Edin. Med. and Surg. Jour.*, July 1840.—*Ibid.*

